# Twelve tips for bringing competencies into continuing professional development: Curriculum mapping

**DOI:** 10.15694/mep.2019.000145.1

**Published:** 2019-07-01

**Authors:** Robert Parson, Natalia Danilovich, Heather Lochnan, Simon Kitto, Dianne Delva, Gary Viner, Eric Wooltorton, Paul Hendry

**Affiliations:** 1 University of Ottawa

**Keywords:** curriculum mapping, competency-based medical education, competency-based curriculum, continuing professional development, family medicine

## Abstract

This article was migrated. The article was marked as recommended.

There is a growing worldwide awareness in the field of health professions education and research that a successful implementation of competency-based medical education (CBME) requires embracing all stages of professional development (from undergraduate, through residency to continuing education). However, despite increased levels of cognizance and even enthusiasm about the importance of the entire continuum for the ultimate goal of improved healthcare, much work still remains as CBME principles are not widely adopted in continuing professional development (CPD). Much has been written about the process of competency-based curriculum development (e.g., the formation and development of meaningful and measurable outcomes) in undergraduate studies and postgraduate training, but not in CPD. If we expect a CPD curriculum to integrate CBME, competencies must be developed and clearly specified how they will fit into a coherent and implementable curriculum structure. In this article, we describe existing practices some educational institutions have, including our experiences in the Office of CPD at the University of Ottawa, Canada, in designing a competency-based curriculum and provide 12 tips for those who begin their journey of organizing, developing, and implementing such curricula. We conclude that in order to translate a competency-based approach into CPD, educational programs will have to refine curricula *across health professionals*’ *education* using curriculum mapping as an important tool of curriculum development and evaluation.

## Introduction

Competency-based medical education (CBME) is an outcomes-based approach to the design, delivery, and evaluation of an educational program using an organized framework of competencies, which ultimately may lead to better care for patients ( Frank *et al.,* 2010a). Promoting a true continuum of medical education from undergraduate, through residency to Continuing Professional Development (CPD) seems to be an ideal scenario to improve quality and patient safety, considering that all stages of professional developmental contribute to and promote physicians’ knowledge, skills, attitudes, and behaviors ( Campbell *et al.,* 2010; Frank *et al.,* 2010a; Lockyer *et al.,* 2017).

Implementing CBME is a time-consuming and complicated process, which starts with the formation and development of meaningful and measurable outcomes and of a competency-based curriculum ( Kern, 1998; Diamond, 2008). It requires ‘translation’ of formulated competencies into intended learning outcomes and assessment formats ( Koster, Schalekamp and Meijerman, 2017). These are some of the major challenges to the implementation process ( Caverzagie *et al.,* 2017) which underpins the fact that CBME principles are not widely adopted in CPD ( Lockyer *et al.,* 2017; Nousiainen *et al.,* 2017). As competency-based education is increasingly used across medical education systems worldwide, much of the recent discussion aroundcompetency-based curriculum design and development has focused solely on undergraduate and residency programs ( Frank *et al.,* 2010a; Sherbino *et al.,* 2013; Hawkins *et al.,* 2015; Eva *et al.,* 2016; Tannenbaum *et al.,* 2011; Ross *et al.,* 2011; Allen *et al.,* 2011; Iglar, Whitehead and Takahashi, 2013; Lacasse, Théorêt and Tessier, 2014; McEwen, Griffiths and Schultz, 2015; Schultz and Griffiths, 2016; Edwards and Frey, 2007; Tudiver *et al.,* 2009; Jansen and Rosenbaum, 2016; Thomas and Kreptul, 2015). Lately, our team has also demonstrated that research on competency-based CPD, family medicine in particular, is quite limited ( Kitto *et al.,* 2018). If we expect a CPD curriculum for healthcare practitioners to integrate CBME, competencies must be defined and present in the curriculum. To gain a better insight into and develop a better grasp of curriculum content (e.g., to what extent competencies were addressed in the curriculum), a curriculum evaluation has to be performed ( Muntinga *et al.,* 2016). One of the common strategies in evaluating the competencies specified in a curriculum is *curriculum mapping*, which refers to “the system that allows content to be mapped across the curriculum and adjusted to minimize gaps and unnecessary redundancies” ( Thomas *et al.,* 2015, p. 218]. Curriculum mapping shows how and when content is taught, and what outcomes are measured to determine success ( Harden, 2001).

Based on current evidence and the authors’ experiences in the Office of CPD at the University of Ottawa, Canada, we outline 12 practical tips that can be used as a guide by all stakeholders (teaching staff, practitioners, managers, and representatives of the discipline) involved in CPD curriculum development. We argue that a CPD provider organization should concentrate on and employ the principles of a competency-based approach in concert with strategies from the field of quality improvement (QI). We believe that this will bolster the successful implementation of the CPD curriculum and hopefully lead toward better patient outcomes, the latter being the key focus of contemporary CPD systems ( Sargeant, Wong and Campbell, 2018).

The purpose of this paper is to explore the current practices regarding curriculum mapping process in an effort to identify effective ones that could be used in the development of competency-based CPD programs. We will also offer some insights into ways in which those practices might be adapted as a response to the need for reforming health professions education across the continuum. It is important to note that although the program under discussion relates to CPD in family medicine, the broad issues raised in this paper are still transferable to and useful for other disciplines, educational contexts and countries.

## Tip 1 Set up a coordination committee for systematic project management

Before the mapping process starts, the project should be systematically planned, including determining an overall goal(s), communication strategies, and a timeline along with defining the evaluation and success criteria of the mapping project ( Harden, 2001; Lachiver and Tardif, 2002). Stakeholders should be identified, resources allocated and strategies and steps in the process developed (Totté, , Huyghe and Verhagen, date unknown).

Important first steps include identification of a project coordinator, who is familiar with the field and possesses the requisite expert knowledge (curriculum mapping, project management, change management) ( Lammerding-Koeppel *et al.,* 2017); recruitment of an influential supporter/leader (usually dean and/or dean of study), who can promote the importance of the project and support the coordinator ( Lachiver and Tardif, 2002; Willett, 2008); maintenance of a consistent working group membership as much as possible, to maintain the “corporate memory” of the project ( Kelley *et al.,* 2008).

During the academic year 2016-2017, the Vice Dean of CPD (PH) at the University of Ottawa appointed the initial working group to evaluate the Office of CPD’s existing family medicine CPD curriculum in anticipation of an upcoming accreditation survey. The goal was to determine if the program was meeting the needs of the learners and to establish a process to address the needs in the future.

## Tip 2 Consult all your stakeholders

Aligning medical education with the needs of patients and society can only happen if all relevant stakeholders are engaged in determining meaningful and measurable outcomes ( Koster, Schalekamp and Meijerman, 2017; Caverzagie *et al.,* 2017; Sargeant, Wong and Campbell, 2018). These outcomes should be defined for individual trainees and their residency programs (e.g. Royal College of Physicians and Surgeons CanMEDS Roles; Accreditation Council for Graduate Medical Education (ACGME) Milestones Project, Caverzagie *et al.,* (2017) and could potentially be used to guide outcomes for practicing family physicians. Involving all stakeholders from the start of the curriculum mapping process, and focusing on a common goal, such as quality in higher education, appears crucial for curriculum development ( Lachiver and Tardif, 2002; Leisyte *et al.,* 2013; Koster, Schalekamp and Meijerman, 2017).

The University of Ottawa curriculum mapping project included a mixed stakeholder group of experts with different backgrounds and expertise (family medicine, higher education, and CPD) and those representing different organizations (university, community, education organizations). The group included the Vice Dean, CPD (PH), as an expert on CPD as well as the university’s structure and organization, five other family medicine clinician educators, and the Director of Education and Accreditation (RP) from the Office of CPD. The working group benefited from collaborative consultations which ensured a process that was relevant and grounded in solid principles. This strategy provided a unique opportunity to promote a sense of ownership among stakeholders ( Kayyal and Gibbs 2012; Zelenitsky et al. 2014).

## Tip 3 Establish clear communication channels

Clear and regular communication between stakeholders and educators across the system (Dean’s office, departments, planning committees, and teachers) are necessary for an effectual transition to CBME ( Nousiainen *et al.,* 2017, Lammerding-Koeppel *et al.,* 2017). Thus, strategies for clear and regular communications must be elaborated during working group meetings/workshops at the beginning and throughout the curriculum mapping project to avoid misalignment of activities and maintenance of timelines ( Gorrell, Beirman and Vemulpad, 2015; Koster, Schalekamp and Meijerman, 2017). Preserving the institutional memory requires careful and continuous documentation and communication of progress or delays of the mappings process including results of project meetings and committee work (information, discussion, records, etc.) ( Lachiver and Tardif, 2002; Lammerding-Koeppel *et al.,* 2017).

During our working group meetings, communication plans and strategies were defined, and we were able to ensure alignment on definitions of clinical and CanMEDs competencies pertaining to curriculum mapping. Targets, courses and results of meetings were carefully documented. Background work and sharing of documents was performed through email and telephone calls.

## Tip 4 Select or create a competency framework

Devising the “organized framework of competencies” ( Frank *et al.,* 2010a) is one of the most important initial steps in implementing CBME as it determines *what* should be included in a comprehensive competency-based curriculum ( MacDonald *et al.,* 2012; Arafeh, 2015; Koster, Schalekamp and Meijerman, 2017). Several competency-based frameworks have been developed in different countries. The three most widely accepted competency-based frameworks are the CanMEDS model ( Frank, Snell and Sherbino, 2015), the Good Medical Practice standard in the United Kingdom ( General Medical Council, 2013), and the Outcomes Project of the Accreditation Council for Graduate Medical Education in the United States ( Swing, 2007). All can be used as a starting point for curriculum development but interpretation and fine-tuning to the local context is necessary ( Koster, Schalekamp and Meijerman, 2017; McGaghie *et al.,* 1978).

Lockyer *et al.* (2017) suggest that a CBME CPD program requires a competency context framework that encompasses more than simply the clinical abilities, but also integrates the whole range of skills, as exemplified in the CanMEDS roles. However, as a competency framework, CanMEDS is insufficient on its own, due in part to the “abstract and general descriptions of the roles complicating their translation into the realities of day-to-day practice” ( Van der Lee *et al.,* 2013, p. 949). Whereas the framework must encompass a holistic view of family medicine practice, it also has to be tempered. For example, a framework that contained hundreds of competencies would require so much time to map that it could make the curriculum mapping process unattractive or unusable.

Once the complete list of competencies is approved by the expert working group, it has to be published and distributed among all stakeholders (e.g., practitioners, CPD providers, regulators) to make them aware of the expected outcomes. The literature supports the claim that publishing and sharing the competencies is a useful strategy to facilitate education ( Harden, Crosby and Davis, 1999; Palés *et al.,* 2004) as it reminds all stakeholders of their commitment to work towards the overall mission ( Lachiver and Tardif, 2002; Frank *et al.,* 2010b).

The CPD Office adopted the curriculum framework which was developed locally by the family medicine residency program at the University of Ottawa ( MacDonald *et al.,* 2012). The framework is based on:

a. the four principles of family medicine ( Rosser, 2006):

b. the importance of the doctor-patient relationship, the family physician being a skilled clinician, family medicine being a community-based discipline, and the family physician being a resource to a defined practice;

c. the seven CanMEDS-FM roles/competencies ( Tannenbaum *et al.,* 2009):

d. family medicine expert, communicator, collaborator, manager, scholar, health advocate, and professional;

e. the Triple C curriculum principles ( College of Family Physicians of Canada, 2012):

f. comprehensive care, continuous care, and centered in family medicine;

g. the eight curriculum content domains/knowledge areas of family medicine ( McDonald *et al.,* 2012):

h. maternity/newborn, children/adolescence, adults, elderly, end-of-life care, procedural skills, behavioural medicine/mental health/ethics, and special populations;

i. the pedagogic strategies, all of which support the development of attitudes, knowledge, and skills in family medicine CPD training ( MacDonald *et al.,* 2012).

The framework was then reviewed by team members and adapted to reflect the realities of practicing family medicine practitioners.

## Tip 5 Formulate the questions that should be answered about the curriculum

When embarking on the process of mapping a curriculum it is essential to decide what questions should be answered about the curriculum ( Denny *et al.,* 2005; Kelley *et al.,* 2008). An important consideration in this step of creating the curricular questions is addressing any required (accreditation-related) questions. For example, “What types of teaching and learning strategies are used in the delivery of the curriculum? To what extent do particular courses contribute to a particular outcome? Where do we teach what? What teaching methods are used in each course? How do we measure what students learn relative to each of these outcomes?” The answers depend on the extent of the reform/curricular change, its nature, and the local context ( Lachiver and Tardif, 2002).

The CPD Office at the University of Ottawa wanted to review the current family medicine curriculum with the intent to answer the following questions: Does our family medicine CPD curriculum cover all of the expected competencies of a practicing family medicine physician? Which modules/courses contribute to the accomplishment of each of the competencies?

## Tip 6 Use curriculum mapping as a tool for continuous quality improvement

As the purposes and motivations for engaging in curriculum mapping may be numerous, it is important from the beginning to define the purpose of using this tool is. There has been interest in the use of curriculum maps in health professions education for their potential application in program evaluation to promote continuous quality improvement ( Harden, 2001; Plaza *et al.,* 2007; Willett, 2008; Kayyal and Gibbs, 2012; Zelenitsky *et al.,* 2014; Gorrell, Beirman and Vemulpad, 2015; Malone *et al.,* 2015; Rawle *et al.,* 2017). The tool has been extensively used in higher education to articulate and revise an academic curriculum ( Arafeh, 2015; Kapucu, 2017), to visualize curricular goals, scopes, and outcomes ( Maki, 2001; DeLuca and Bellara, 2013), to track curricular changes over time ( Kelley *et al.,* 2008) and to assess a program’s ability to address professional accreditation requirements ( Kelley *et al.,* 2008; Perlin, 2011; Veltri *et al.,* 2011). Mapping the various curricular elements against existing frameworks can be very helpful in identifying actual or potential deficiencies, overlaps, inconsistencies, discontinuities, and areas for improvement in the curriculum through consultation of different stakeholders ( Willett, 2008; Totté, Huyghe and Verhagen, [date unknown]; Koster, Schalekamp and Meijerman, 2017). Successful implementation of curriculum mapping is particularly important, because with the approval of the CanMEDs, it is advisable for the faculties to restructure the curriculum in a competence-oriented way ( Tannenbaum *et al.,* 2011). And, in order to ensure that planned outcomes are achieved effectively and efficiently, any curriculum transformation should incorporate aspects of quality assurance ( Kayyal and Gibbs, 2012).

## Tip 7 Create curriculum mapping tool and the data-collection methods

There are several techniques used to map a curriculum, and traditionally, several stages are involved ( Harden, 2001; Prideaux, 2003; Sumsion and Goodfellow, 2004; Diamond, 2008; Uchiyama and Radin, 2009; Ervin, Carter and Robinson, 2013), which is beyond the scope of this paper. No one approach seems ideal, but it is possible that one approach (or a combination of several) could best suit your own needs. O’Neill (2015) recommends some considerations that may help in the selection of the curriculum mapping techniques might best suit your needs. You have to decide a) the purposes of the curriculum mapping, b) who and how many people will be involved in the mapping, c) the timing of the mapping in the curriculum design process, and d) how the information will be used and shared. No one approach seems ideal, but it is possible that one approach (or a combination of several) could best suit your own needs.

Once the competency framework and the questions have been developed, the next step consists of identifying the right metrics for the data gathering phase ( Kelley *et al.,* 2008; Willett, 2008), specifying the sequence of the mapping exercise, and establishing a common understanding of terms and processes ( Rawle *et al.,* 2017). These consistent definitions are important for maintaining interrater reliability among the faculty members who will be mapping the outcomes (or content) to their courses ( Kelley *et al.,* 2008).

While assessing the program at the University of Ottawa, we used the existing curriculum (University of Ottawa CPD Conferences for Family Medicine, 2015-2017) and course syllabi as evidence to map learning outcomes ( Sumsion and Goodfellow, 2004; Cuevas, Matveev and Miller, 2010; Veltri *et al.,* 2011; Arafeh, 2015). This technique, among other details, included the learning outcomes from each learning session, that were then mapped to the relevant competency(ies) (see Tip 8).

## Tip 8 Conduct a pilot study

For the mapping instrument to be acceptable, it is very important that it must be coder-friendly and meet the coders’ expectations ( Lammerding-Koeppel *et al.,* 2017). Therefore, conducting a “pilot study” can be helpful ( Kelley *et al.,* 2008; Lammerding-Koeppel *et al.,* 2018).

In our pilot study,the curriculum map matrix was initially tested by two members of the expert steering committee (PH and HL) who evaluated/mapped one CPD program/event (Update in Cardiology, 2015) made up of 13 sessions. This was mapped to our preliminary competency framework. As a result of this first step, a new category/domain *“Practitioner’s Category”* was incorporated into the competency-based framework. The new category/domain contained the following four competencies:

• Practice quality improvement (QI) and patient safety
• Manage resources, such as community services and external resources, efficiently
• Recognize, and respond to, the patient experience
• Practice a process or stringent review of actual medical needs for tests and procedures (i.e.: Choosing Wisely)

As a second step, to further assure quality and consistency, the expert steering committee selected two CPD events that were mapped by all its members. The coders worked independently and later each coder submitted his/her set of coding results. Their results were compared and discussed during the meetings to arrive at agreement and consistency.

Based on input from the coders’ experience with the pilot study, the original mapping form was revised to reduce the number of the mapping categories. The literature also stressed the importance of the adjustment (reduce/expand) of the mapping categories from the originally planned categories ( Harden, 2001; O’Neill, 2015; Lammerding-Koeppel *et al.,* 2017). We decided to use Excel for all data entry. The completed curriculum matrix consisted of two elements: (1) learning events, such as lectures and workshops from conferences and (2) learning outcomes/competencies framework (which included the Family Medicine competency list, the CanMeds framework and the new category/domain *“Practitioner’s Category.”* Overall, 100 competencies were listed in the competency framework. We analyzed 24 conferences developed over the last 2 years. Over 1,000 learning objectives from 495 separate sessions, lectures or workshops were mapped to the competencies.

## Tip 9 Anticipate barriers and difficulties

The curriculum mapping process may be challenging to those involved with CPD development. Implementing a competency-based CPD curriculum will not come without significant challenges. One of the key challenges in implementing such change is overcoming resistance to change ( Gale and Grant, 1997; Dale, 2007). There is an expectation for faculty to participate in the process that requires time which is always in short supply in medical education contexts. Consideration of what has been covered in the past and what gaps are currently present will have to be entertained by CPD developers using the matrix as a guide.

Every professional environment requires collaboration and adaptability to change in order to maximize success ( Dale, 2007). Therefore, the purpose and benefits of curriculum mapping should be made clear to all stakeholders as a way of displaying and reviewing the CPD curriculum and as a resource and guide in curriculum development, not a prescription or a series of boxes that must be checked ( Kelley *et al.,* 2008).

## Tip 10 Stimulate faculty involvement and development

Curriculum development and mapping are considered a bottom-up approach with faculty engagement and development being critical ( Williams and Bolland, 2011). Faculty (Scientific Planning Committee in CPD) participation improves the consistency and reliability of data used to construct the curriculum map ( Zelenitsky *et al.,* 2014; Lammerding-Koeppel *et al.,* 2017). Therefore, successful curriculum mapping implementation requires trained staff for coordination, while the institutional management structure should support the required human resources allocation ( Jacobs *et al.,* 2005; Koster, Schalekamp and Meijerman, 2017).

## Tip 11 Ensure leadership commitment to change

A key factor in determining QI success is top management’s commitment towards quality management ( Lakshman, 2006; Dale, 2007). Similarly, successful implementation of curriculum mapping in CPD requires management commitment and appropriate leadership that not only communicate and promote the shared vision of curricular change, but also are able to obtain appropriate resources ( Bland *et al.,* 2000; Zelenitsky *et al.,* 2014). This type of support reinforces the importance and value of the curriculum mapping project and helps to ensure follow-through ( Zelenitsky *et al.,* 2014). Given that committed involvement is a challenge, due to the time-consuming and longitudinal nature of curriculum mapping ( Kapucu, 2017), the appropriate leadership attracts individuals to a rallying objective defined by a mission and sustains a climate of confidence that drives the determination to succeed ( Lachiver and Tardif, 2002).

At the University of Ottawa, from the start, the Vice Dean of CPD (PH) has been actively engaged in the curriculum mapping initiative. The inclusion of a key decision maker, such as Vice Dean CPD, has been crucial for the implementation of the decisions.

## Tip 12 Develop an implementation plan for curriculum change

Once competencies are identified, the course objectives are mapped to the competencies, and the gaps in the curriculum are determined, the members of the expert committee need to develop an implementation plan for any expected curriculum changes or improvements ( Kelley *et al.,* 2008). It ensures that the map is part of a process of continuous curricular improvement and not simply an exercise in data gathering. Implementing the plan does not assure success or reduce the obstacles arising from resistance to change, but it can remind all stakeholders of the common goal underlying the project and provide tools for assessing new practices ( Lachiver and Tardif, 2002).

## Conclusion

In this paper, we described the essential steps in designing a competency-based CPD curriculum and offer tips for a successful organization, development, and implementation of such curricula. The suggestions stem from relevant literature as well as the authors’ own experiences of implementing a competency-based CPD curriculum in the field of family medicine.

Effective CPD that is based on the competency framework and emphasizes quality improvement efforts during the practice years is vital for all healthcare providers as it promotes life-long learning, practice-based change, and improved clinical outcomes ( Kitto *et al.,* 2013; Sargeant, Bruce and Campbell, 2013; Eva *et al.,* 2016; Lockyer et al., 2017). While CMBE has been adopted in family medicine training programs, it has yet to be adopted by the CPD community.

The CPD program at the University of Ottawa sought to fill this gap. The current curriculum mapping initiative is still in its early stages, but we have identified some omissions, inconsistencies, and areas for improvement, that we hope will guide us in developing a comprehensive competency-based curriculum for family physicians. The eventual anticipated goal of the curriculum mapping in family medicine CPD project is to create concepts that are transferrable to other medical settings to inform the design and implementation of CBME in CPD. We anticipate that the results of the curriculum mapping project, will be immediately relevant for CPD programs in family medicine as well as to CPD programs across medicine more generally.

Future efforts will focus on improving the implementation and evaluation of the curriculum. One of the important issues to settle is how to make a smooth transition from the old to the new curriculum. We believe that curriculum mapping is a useful tool in supporting a competency-based family medicine CPD program and is a step along the way to improve our understanding about how CPD can evolve for the future.

## Take Home Messages

• While CMBE has been adopted in residency training programs, it has yet to be adopted by the CPD community.
• In order to translate a competency-based approach into CPD, educational programs will have to refine curricula *across health professionals*’ *education* using curriculum mapping as an important tool of curriculum development and evaluation.
• The eventual anticipated goal of the curriculum mapping in family medicine CPD project is to create concepts that are transferrable to other medical settings to inform the design and implementation of CBME in CPD.

## Notes On Contributors

**Robert Parson,** MEd,Director of Education and Accreditation in the Office of Continuing Professional Development at the University of Ottawa.

**Dr Natalia Danilovich**, MD, PhD, Research Associate in the Office of Continuing Professional Development and the Department of Innovation in Medical Education at the University of Ottawa.

**Dr Heather Lochnan**, MD, FRCPC, the Assistant Dean of Continuing Professional Development, Education Programming for the Faculty of Medicine, an Endocrinologist and Associate Professor in the Department of Medicine at the University of Ottawa.

**Dr Simon Kitto**, PhD, Director of Research at the Office of Continuing Professional Development and Professor in the Department of Innovation in Medical Education at the University of Ottawa.

**Dr Dianne Delva,** MD, CCFP, FCFP, Chair and Professor in the Department of Family Medicine at the University of Ottawa.

**Dr Gary Viner,** MD, Director of Evaluation in Postgraduate Program and Associate Professor in the Department of Family Medicine at the University of Ottawa.

**Dr Eric Wooltorton,** MD, Director of Faculty Development and Assistant Professor in the Department of Family Medicine at the University of Ottawa.

**Dr Paul Hendry**, MD, MSc, FRCSC, Vice-Dean of Continuing Professional Development in the Faculty of Medicine at the University of Ottawa. He is a Cardiac Surgeon at the University of Ottawa Heart Institute and Professor of Surgery at the University of Ottawa.
