# Supplementary material for: Twelve tips for bringing competencies into continuing professional development: Curriculum mapping
Source: MedEdPublish (2016). 2019 Jul 1;8:145. [Version 1] doi: 10.15694/mep.2019.000145.1 (PMC10911764; doi:10.15694/mep.2019.000145.1)
Supplement: Peer review history

# Reviewer report: Reviewer response for version 1

**DOI:** 10.21956/mep.19870.r30030

**Published:** 2019-07-14

**Authors:** Helena Filipe

**Affiliations:** 1 Hospital of the Armed Forces/PL-EMGFA

This review has been migrated. The reviewer awarded 5 stars out of 5 Authors are highly skillful in competency based CPD and renowned by their vast experience in CPD scholarship. Successful implementation of CMBE involves covering all stages of professional development from undergraduate, residency extending to CPD. Recognizing that this method has not yet been widely adopted in CPD, they summarize their experience into 12 evidence based practical recommendations, useful for CPD curriculum developers. Curriculum mapping is emphasized as the master tool to effectively translate continuing education into the development and implementation of meaningful and measurable outcomes. The concept underlying curriculum mapping and the process to undertake this curriculum development approach are comprehensively explained. The reader is compelled to read the article in one fell swoop from tip to tip, so elegant, clear, concise and fluid the writing is. This guidelines model can be replicated and adapted into different settings and different medical specialties.The 12 stepwise guidelines could highlight 12 keywords to keep in mind during the process of developing a curriculum in CPD: Plan, Include, Communicate, Create, Formulate, Revise, Map, Pilot, Anticipate, Attract, Manage and Implement.

Reviewer Expertise:

NA

No decision status is available

# Reviewer report: Reviewer response for version 1

**DOI:** 10.21956/mep.19870.r30032

**Published:** 2019-07-03

**Authors:** BALAJI ARUMUGAM

**Affiliations:** 1 TAGORE MEDICAL COLLEGE AND HOSPITAL

This review has been migrated. The reviewer awarded 4 stars out of 5 GOOD WORK BY THE TEAM OF AUTHORS. FELT HAPPY AND GREAT TO READ SUCH A CLEAR AND CONCISE ARTICLE ON TIPS FOR CBME--> CPD WITH CURRICULUM MAPPING. This article speaks about 1. CBME principles are not widely adopted in continuing professional development (CPD)2. If we expect a CPD curriculum to integrate CBME, competencies must be developed and clearly specified how they will fit into a coherent and implementable curriculum structure.3. curriculum mapping, which refers to “the system that allows content to be mapped across the curriculum and adjusted to minimize gaps and unnecessary redundancies” WHICH MAY ALSO BE CALLED AS ALIGNED AND INTEGRATED CURRICULUM.....SUGGESTION FROM THE REVIEWER ARE..... 4. Consult all your stakeholders and sensitize them with the curriculum mapping, (Alignment and integration) can be the first step and then the setting up the coordination committee with subcommittees can be the second step. 5. Quality control team (committee for curriculum mapping) can be another important tip after the implementation process to cross check any deviations from the mapped curriculum.

Reviewer Expertise:

NA

No decision status is available

# Reviewer report: Reviewer response for version 1

**DOI:** 10.21956/mep.19870.r30031

**Published:** 2019-07-02

**Authors:** Augusto Scalabrini Neto

**Affiliations:** 1 University of Sao Paulo School of Medicine

This review has been migrated. The reviewer awarded 5 stars out of 5 This is a very interesting guideline on curriculum development and CBE. Well written, clear and practical, will be very useful to people who seek CD using CBE.

Reviewer Expertise:

NA

No decision status is available
